# Continuous Glucose Monitoring for the Diagnosis of Post-Transplantation Diabetes Mellitus and Impaired Glucose Tolerance From Years One to Five After Kidney Transplantation—A Prospective Pilot Study

**DOI:** 10.3389/ti.2024.13724

**Published:** 2024-11-15

**Authors:** Georgios Eleftheriadis, Marcel G. Naik, Bilgin Osmanodja, Lutz Liefeldt, Fabian Halleck, Mira Choi, Eva Schrezenmeier, Bianca Zukunft, Andrea Tura, Klemens Budde

**Affiliations:** ^1^ Department of Nephrology and Medical Intensive Care, Charité—Universitätsmedizin Berlin, Corporate Member of Freie Universität Berlin and Humboldt-Universität zu Berlin, Berlin, Germany; ^2^ CNR Institute of Neuroscience, Padova, Italy

**Keywords:** kidney transplantation, cardiovascular disease, Post-transplantation diabetes mellitus, prediabetes, continuous glucose monitoring

## Abstract

Post-transplantation diabetes mellitus (PTDM) and prediabetes are associated with increased cardiovascular morbidity and mortality in kidney transplant recipients (KTR), when diagnosed by an oral glucose tolerance test (oGTT). Hemoglobin A1c (HbA1c) and fasting plasma glucose (FPG) display low concordance with the oGTT in the early phase posttransplant. For this prospective cross-sectional pilot study, 41 KTR from years one to five after transplantation without known preexisting PTDM (defined by HbA1c ≥ 6.5% (NGSP) or 48 mmol/mol (IFCC) at last visit or glucose-lowering therapy) were recruited at the Charité Transplant Outpatient Clinic. For each study participant HbA1c, FPG and an oGTT were followed by CGM. 38 of the 41 patients recruited had sufficient CGM-recordings (≥10 days). PTDM and impaired glucose tolerance (IGT), as defined by the gold standard oral glucose tolerance test (oGTT)-derived 2-h plasma glucose (2hPG), were diagnosed in one (3%) and twelve (32%) patients, respectively. HbA1c exhibited good test characteristics regarding IGT (ROC-AUC: 0.87); sensitivity/specificity of HbA1c-threshold 5.7% (NGSP) or 39 mmol/mol (IFCC) were 1.0/0.64, respectively. Best performing CGM-readouts mean sensor glucose and percent of time >140 mg/dL (%TAR (140 mg/dL)) displayed acceptable diagnostic performance (ROC-AUC: 0.78 for both). Thus, HbA1c can aid in timely diagnosis of IGT in the stable phase after kidney transplantation.

## Graphical Abstract

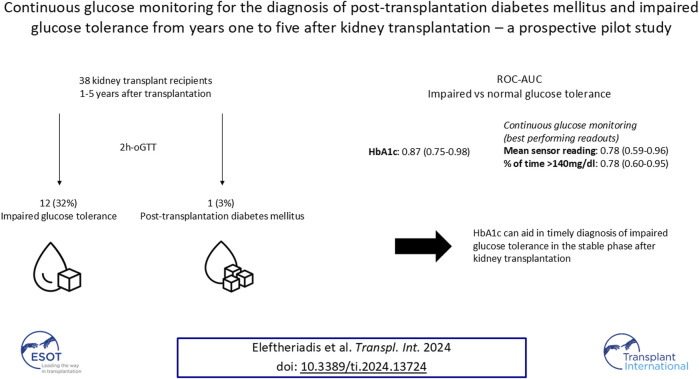

## Introduction

Post-transplantation diabetes mellitus (PTDM) and prediabetes affect 20%–30% of kidney transplant recipients (KTR) and are associated with increased cardiovascular morbidity and mortality, when diagnosed by an oral glucose tolerance test (oGTT) [1–3]. Though widely regarded as the gold standard for the diagnosis of PTDM and prediabetes [4, 5], routine implementation of the oGTT is impeded by its time consuming and impractical nature in most large transplant programs [4]. Pathophysiologic alterations in the early stage posttransplant, in particular increased rates of red blood cell turnover, immunosuppressive effects on erythrocyte proliferation in the bone marrow and steroid-induced glucose maxima in the early afternoon and evening, contribute to a severely compromised validity of hemoglobin A1c (HbA1c) and fasting plasma glucose (FPG) during this stage [6–8]. In fact, neither HbA1c nor FPG in the first year after kidney transplantation show a robust association with patient survival or cardiovascular events [2, 3, 9]. Test characteristics of HbA1c and FPG have been shown to improve in the second year after kidney transplantation compared to the gold standard oGTT, though still remaining suboptimal [10, 11]. Concordance of glycemic parameters >2 years after kidney transplantation has not been extensively studied.

Continuous glucose monitoring (CGM) has transformed diabetes care for patients with diabetes mellitus type 1 and 2, improving glycemic management and lowering the risk of acute diabetic complications and hospital admissions [12, 13]. Experience of CGM-utilization after kidney transplantation has been limited [8, 14–17], especially with regards to the stable phase (>1 year) after transplantation [16].

The aim of this prospective cross-sectional pilot study was to assess feasibility of CGM and investigate its potential for the diagnosis of PTDM and IGT based on the gold standard oral glucose tolerance test (oGTT)-derived 2-h plasma glucose (2hPG) in patients without known preexisting PTDM one to 5 years after kidney transplantation.

## Patients and Methods

### Study Design

**FIGURE 1:**
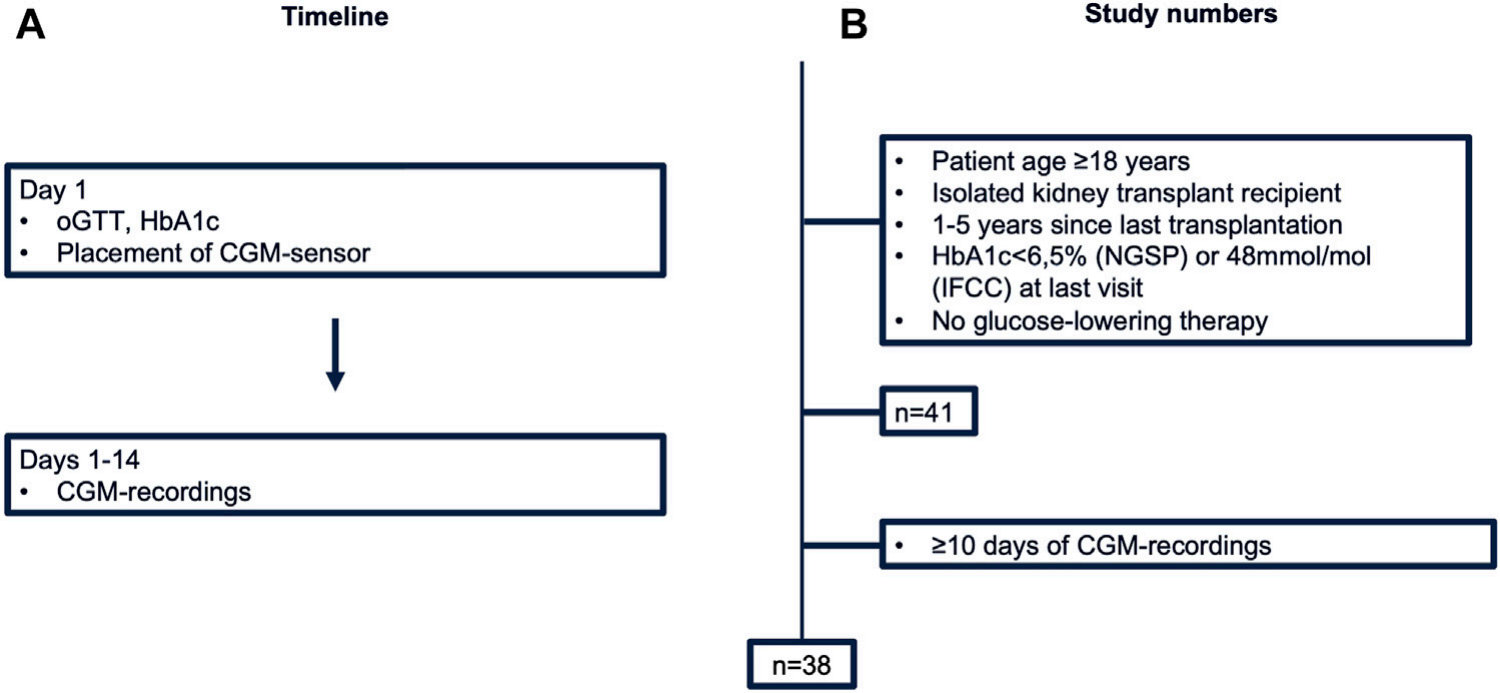
Study design **(A)** and numbers **(B)**. CGM, continuous glucose monitoring; HbA1c, hemoglobin A1c; IFCC, International Federation of Clinical Chemistry and Laboratory Medicine; NGSP, National Glycohemoglobin Standardization Program; oGTT, oral glucose tolerance test.

This prospective cross-sectional pilot study was conducted between September 2022 and May 2023 at our Transplant Center at the Department of Nephrology and Medical Intensive Care, Charité – Universitätsmedizin Berlin. Study design and study numbers are shown in Figure 1. Inclusion criteria were: (i) age ≥18 years (ii) isolated kidney transplant recipient (iii) one to 5 years since last transplantation. Patients with known PTDM (diagnosed through HbA1c ≥ 6.5% (NGSP) or 48 mmol/mol (IFCC) at last visit or glucose-lowering therapy) were excluded from the study. The study protocol was approved by the Ethics Committee of Charité – Universitätsmedizin Berlin (EA4/110/22). All evaluations were performed according to the Declaration of Helsinki (2013 Amendment). Written informed consent was obtained from each participant.

### Laboratory Measurements

Blood tubes were sent to the laboratory for analysis directly after blood drawing. HbA1c (ethylenediamine tetraacetic acid tube) was measured by highperformance liquid chromatography separation of hemoglobin fractions. An oral glucose tolerance test (oGTT), consisting of a glucose load containing the equivalent of 75 g anhydrous glucose dissolved in water as described by the WHO, was performed with blood drawings at timepoints 0, 1 h and 2 h [4]. FPG was obtained as part of the oGTT. Plasma glucose (sodium fluoride tube) was assessed by the hexokinase method.

### Diagnostic Criteria for PTDM and IGT

Diagnosis of PTDM and IGT was based on the 2hPG-criterion of the American Diabetes Association (ADA) (Supplementary Table S1) [18]. PTDM was defined by oral glucose tolerance test-derived 2-h plasma glucose (2hPG) ≥200 mg/dL, IGT by 2hPG ≥ 140 mg/dL in the absence of PTDM and normal glucose tolerance (NGT) by 2hPG < 140 mg/dL. Index test results were not available to the assessors of the reference standard.

### CGM Recordings

Continuous Glucose Monitoring (CGM) was performed with the “FreeStyle Libre Pro IQ Sensor” (Abbott GmbH, Wiesbaden, Germany). Sensors were placed on the back of the upper arm, with glucose readings blinded for participants and staff. Each sensor was worn for the duration of 14 days and interstitial glucose levels were measured in 15-min intervals. Sensors with ≥10 days recording duration were considered for further analysis [19].

Sensor data were extracted using the “FreeStyle Libre Pro IQ Reader” (Abbott GmbH, Wiesbaden, Germany). CGM files were cleaned and analyzed using the R-package “cgmanalysis” (Version 2.7.7) [20]. The endings of the CGM raw files were trimmed to ensure discrete 24-h chunks. Selection of CGM-readouts was based on the “Recommendations from the International Consensus on Time in Range” [19]. CGM-readouts consisted of: mean sensor readings, percent of time >140 mg/dL [%TAR (140 mg/dL)], percent of time >180 mg/dL [%TAR (180 mg/dL)], percent of time <70 mg/dL [%TBR (70 mg/dL)], estimated A1c, glucose management indicator (GMI), standard deviation (SD), coefficient of variation (CV), low blood glucose index (LBGI), high blood glucose index (HBGI), mean amplitude of glycemic excursions (MAGE) and continuous overall net glycemic action (CONGA) [19]. Reference standard results were not available to the readers of the index test.

### Statistical Analyses

Categorical outcomes were described using frequencies and proportions, while continuous variables were described using means ± standard deviations (SD) or medians and interquartile ranges (IQR) when appropriate. Receiver operating characteristic (ROC) curves for IGT vs. NGT based on the gold standard 2hPG were plotted and the area under the curve (AUC) with respective 95% confidence intervals (CI) calculated. Exploratory screening thresholds for CGM-readouts were based on a sensitivity of around 90% for IGT vs. NGT. Sensitivity, specificity, positive and negative predictive values with 95% CIs, as well as true positives/false negatives and true negatives/false positives for respective IGT thresholds, were calculated. A formal sample size calculation was not performed due to the exploratory design of the study. Patient information was retrieved from our electronic health record and research database for KTR “TBase” [21]. Statistical analysis was performed with “R” version 4.3.1.

We used the Standards for the Reporting of Diagnostic Accuracy Studies (STARD) statement to ensure completeness of reporting [22].

## Results

### Patient Characteristics

41 KTR fulfilled the inclusion criteria and consented to participate. Of these, three patients were excluded from the final analysis due to insufficient CGM-recordings (<10 days). Thus, a total of 38 patients represented the final study population (Table 1). In brief, median age of study participants was 57 years [52–63 years] and 71% (27/38) were male. Median time since last transplant was 3.2 years [1.3 years–4.1 years]. Median eGFR (by CKD-EPI) was 55 mL/min [49–67 mL/min] and urine protein creatinine ratio 87 mg/g [68–110 mg/g]. Primary cause of end stage kidney disease (ESKD) was glomerulonephritis (47%, 18/38), followed by autosomal dominant polycystic kidney disease (ADPKD) (16%, 6/38), while 29% of patients (11/38) reached ESKD without defined underlying cause. 92% (35/38) had one kidney transplant, 42% (16/38) from a living donor. All patients were on calcineurin inhibitor therapy (37/38 tacrolimus, 1/38 ciclosporin), 95% (36/38) received mycophenolate and 89% (34/38) systemic steroid. Patients diagnosed with IGT were older [65 (61,67) vs. 55 (47, 58) years for NGT-patients]. Metabolic (LDL, HDL, total cholesterol, triglycerides) and kidney laboratory parameters (eGFR, UPCR, and UACR) showed overlapping interquartile ranges between groups (Table 2).

**TABLE 1:** Baseline characteristics of study participants.

Characteristic	N = 38^a^
Demographics	
Age (years)	57 (52, 63)
Female/Male	11/27 (29%/71%)
BMI (kg/m2)	25.4 (22.6, 29.2)
Metabolic parameters	
LDL (mg/dL)	106 (75, 132)
HDL (mg/dL)	51 (43, 66)
Total Cholesterol (mg/dL)	183 (148, 224)
Triglycerides (mg/dL)	129 (105, 188)
Kidney parameters	
eGFR (by CKD-EPI, mL/min)	55 (49, 67)
UPCR (mg/g)	87 (68, 110)
UACR (mg/g)	13 (4, 27)
Kidney history	
Number of kidney transplants	
1/2/3	35/2/1 (92%/5%/3%)
Time since last transplantation (years)	3.2 (1.3, 4.1)
DD/LD	22/16 (58%/42%)
Primary cause of ESKD	
Glomerulonephritis	18 (47%)
ADPKD	6 (16%)
Other	3 (7.9%)
Unknown	11 (29%)
Immunosuppression	
Tacrolimus	37 (97%)
Ciclosporin	1 (2.6%)
Mycophenolate	36 (95%)
Systemic steroid	34 (89%)

ADPKD, autosomal dominant polycystic kidney disease; BMI, body mass index; DD, deceased donor; ESKD, End-Stage Kidney Disease; LD, living donor.

^a^ Median (IQR); n (%).

**TABLE 2:** Characteristics of study participants, grouped by 2hPG.

Characteristic	NGT, N = 25^a^	IGT, N = 12^a^
Demographics		
Age (years)	55 (47, 58)	65 (61, 67)
Female/Male	3/22 (12%/88%)	7/5 (58%/42%)
BMI (kg/m2)	25.0 (22.4, 28.1)	25.7 (22.9, 31.0)
Metabolic parameters		
LDL (mg/dL)	103 (76, 131)	103 (63, 134)
HDL (mg/dL)	48 (41, 57)	65 (51, 71)
Total Cholesterol (mg/dL)	191 (147, 225)	181 (147, 222)
Triglycerides (mg/dL)	119 (99, 214)	148 (111, 171)
Kidney parameters		
eGFR (by CKD-EPI, mL/min)	55 (50, 64)	59 (46, 70)
UPCR (mg/g)	84 (59, 109)	97 (81, 192)
UACR (mg/g)	10 (4, 27)	19 (11, 35)
Kidney history		
Number of kidney transplants		
1/2/3	22/2/1 (88%/8%/4%)	12/0/0 (100%/0/0)
Time since last transplantation (years)	3.1 (1.3, 4.0)	4.2 (1.2, 4.8)
DD/LD	13/12 (52%/48%)	8/4 (67%/33%)
Primary cause of ESKD		
Glomerulonephritis	10 (40%)	7 (58%)
ADPKD	4 (16%)	2 (17%)
Other	3 (12%)	0 (0%)
Unknown	8 (32%)	3 (25%)
Immunosuppression		
Tacrolimus	24 (96%)	12 (100%)
Ciclosporin	1 (4.0%)	0 (0%)
Mycophenolate	24 (96%)	11 (92%)
Systemic steroid	22 (88%)	11 (92%)

2hPG, oral glucose tolerance test (oGTT)-derived 2-h plasma glucose; ADPKD, autosomal dominant polycystic kidney disease; BMI, body mass index; DD, deceased donor; ESKD, End-Stage Kidney Disease; IGT, impaired glucose tolerance; LD, living donor; NGT, normal glucose tolerance.

^a^ Median (IQR); n (%).

### Prevalence of PTDM and IGT

**FIGURE 2:**
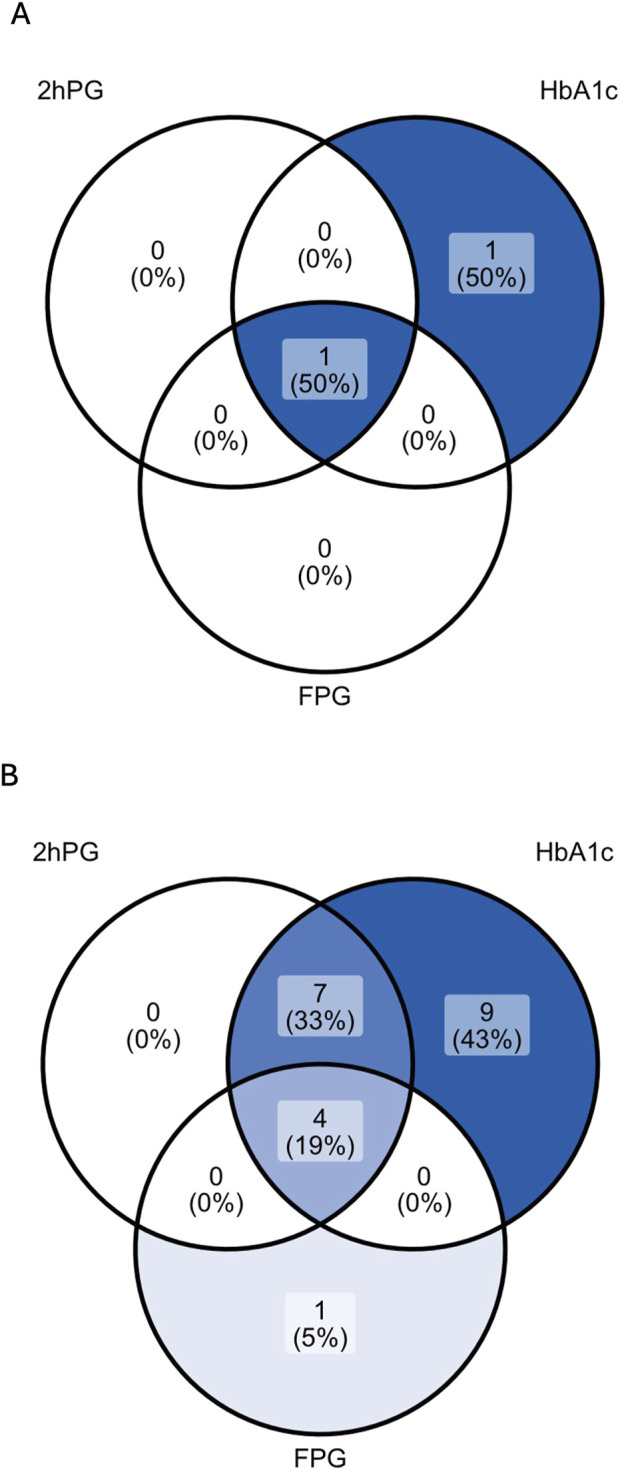
Venn diagrams showing the rate of patients with PTDM **(A)** and prediabetes **(B)** as diagnosed by 2hPG, HbA1c and FPG in patients with complete diagnostic test data. For the analysis of prediabetes **(B)**, patients diagnosed with PTDM by any glycemic test were excluded (n = 2). 2hPG, oGTT-derived 2-h plasma glucose; HbA1c, hemoglobin A1c; FPG, fasting plasma glucose.

Among 38 patients with an oGTT, 3% (1/38) fulfilled the diagnostic criterion of PTDM and 32% (12/38) of IGT by 2hPG. Results of each glycemic test are depicted in Table 3; Figure 2.

**TABLE 3:** Results of glycemic tests.

Glycemic Test	Normoglycemia	Prediabetes	PTDM
*2hPG*	25	12	1
*HbA1c*	16	20	2
*FPG*	31	6	1

Results are shown for patients with all three diagnostic tests.

FPG, fasting plasma glucose; HbA1c, hemoglobin A1c; 2hPG, oral glucose tolerance test (oGTT)-derived 2-h plasma glucose; PTDM, posttransplant diabetes mellitus.

### HbA1c, FPG, and CGM-Readouts

**FIGURE 3:**
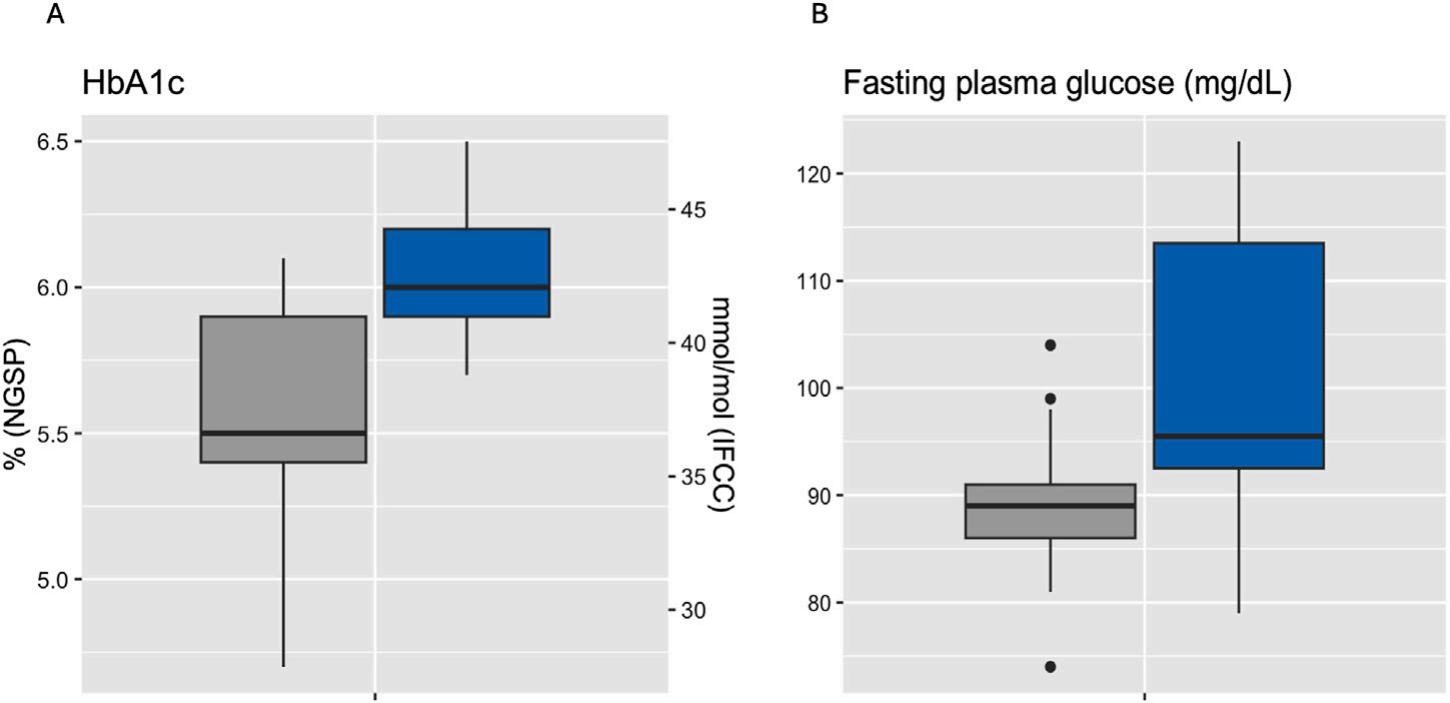
Boxplots of HbA1c **(A)** and fasting plasma glucose **(B)** grouped by 2hPG (*NGT, gray, IGT, blue*). NGT (n = 25), IGT (n = 12). 2hPG, oGTT-derived 2-h plasma glucose; HbA1c, hemoglobin A1c; IFCC, International Federation of Clinical Chemistry and Laboratory Medicine; IGT, impaired glucose tolerance; NGSP, National Glycohemoglobin Standardization Program; NGT, normal glucose tolerance.

**FIGURE 4:**
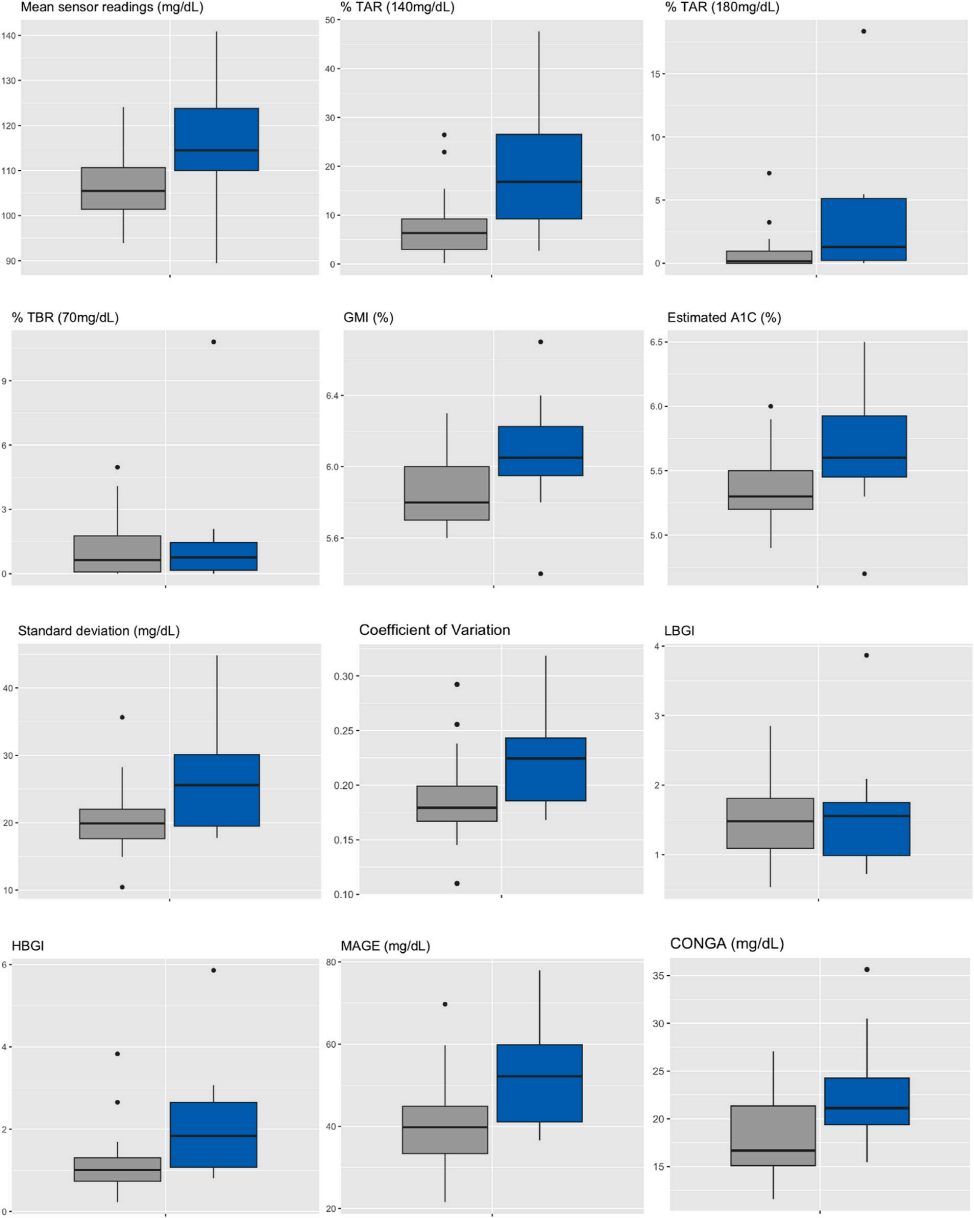
Boxplots of CGM-readouts, grouped by 2hPG (*NGT, gray, IGT, blue*). NGT(n = 25), IGT (n = 12). %TAR, percent of time above range; %TBR, percent of time below range; 2hPG, oral glucose tolerance test (oGTT)-derived 2-h plasma glucose; CONGA, continuous overall net glycemic action; CV, coefficient of variation; GMI, glucose management indicator; HBGI, high blood glucose index; IGT, impaired glucose tolerance; LBGI, low blood glucose index; MAGE, mean amplitude of glycemic excursions; NGT, normal glucose tolerance; SD, standard deviation.

Median HbA1c was 6.0% (NGSP) or 42 mmol/mol (IFCC) [5.9%–6.2% or 41–44 mmol/mol] for IGT-patients and 5.5% (NGSP) or 37 mmol/mol (IFCC) [5.4%–5.9% or 36–41 mmol/mol] for NGT-patients. Median FPG was 96 mg/dL [93–114 mg/dL] for IGT-patients and 89 mg/dL [86–91 mg/dL] for NGT-patients (Table 4; Figure 3). Boxplots and median [IQR] of CGM-readouts, grouped by 2hPG are depicted in Figure 4; Table 5.

**TABLE 4:** HbA1c and FPG, grouped by 2hPG. Median [IQR].

Group	HbA1c (% - NGSP mmol/mol - IFCC)	FPG (mg/dL)
Overall	5.7 [5.5;6.0] 39 [37;42]	90 [87;96]
NGT	5.5 [5.4; 5.9] 37 [36; 41]	89 [86; 91]
IGT	6.0 [5.9; 6.2] 42 [41; 44]	96 [93; 114]

2hPG, oral glucose tolerance test (oGTT)-derived 2-h plasma glucose; FPG, fasting plasma glucose; HbA1c, hemoglobin A1c; IFCC, international federation of clinical chemistry and laboratory medicine; IGT, impaired glucose tolerance; NGSP, national glycohemoglobin standardization program; NGT, normal glucose tolerance.

**TABLE 5:** CGM-readouts, grouped by 2hPG.

CGM-readouts, grouped by 2hPG. Median [IQR]							
	n()	Mean sensor readings (mg/dL)	% TAR (140mg/dL)	% TAR (180mg/dL)	% TBR (70mg/dL)	Estimated A1C (%)	GMI (%)
Overall	37	108 [103;114]	8.6 [4.2;13.7]	0.4 [0;1.3]	0.6 [0.1;1.8]	5.4 [5.2;5.6]	5.9 [5.8;6.0]
*NGT*	25	105 [101;111]	6.3 [3.0;9.2]	0.2 [0;1]	0.6 [0.1;1.8]	5.3 [5.2;5.5]	5.8 [5.7;6.0]
*IGT*	12	114 [110;124]	16.8 [9.2;26.5]	1.3 [0.2;5.1]	0.8 [0.2;1.5]	5.6 [5.5;5.9]	6.1 [6.0;6.2]

	n()	SD (mg/dL)	CV	LBGI	HBGI	MAGE (mg/dL)	CONGA (mg/dL)
Overall	37	20.6 [17.9;23.5]	0.19 [0.17;0.22]	1.5 [1.1;1.8]	1.1 [0.8;1.5]	40.6 [36.1;49.0]	19.36 [15.46;22.04]
*NGT*	25	19.9 [17.7;22.0]	0.18 [0.17;0.20]	1.5 [1.1;1.8]	1.0 [0.7;1.3]	39.8 [33.4;44.9]	16.66 [15.08;21.33]
*IGT*	12	25.6 [19.5;30.1]	0.22 [0.19;0.24]	1.6 [1.0;1.8]	1.8 [1.1;2.7]	52.2 [41.1;59.8]	21.11 [19.36;24.27]

%TAR, percent of time above range; %TBR, percent of time below range; 2hPG, oral glucose tolerance test (oGTT)-derived 2-h plasma glucose; CONGA, continuous overall net glycemic action; CV, coefficient of variation; GMI, glucose management indicator; HBGI, high blood glucose index; IGT, impaired glucose tolerance; LBGI, low blood glucose index; MAGE, mean amplitude of glycemic excursions; NGT, normal glucose tolerance; SD, standard deviation.

### Test Characteristics of HbA1c, FPG, and CGM-Readouts

**FIGURE 5:**
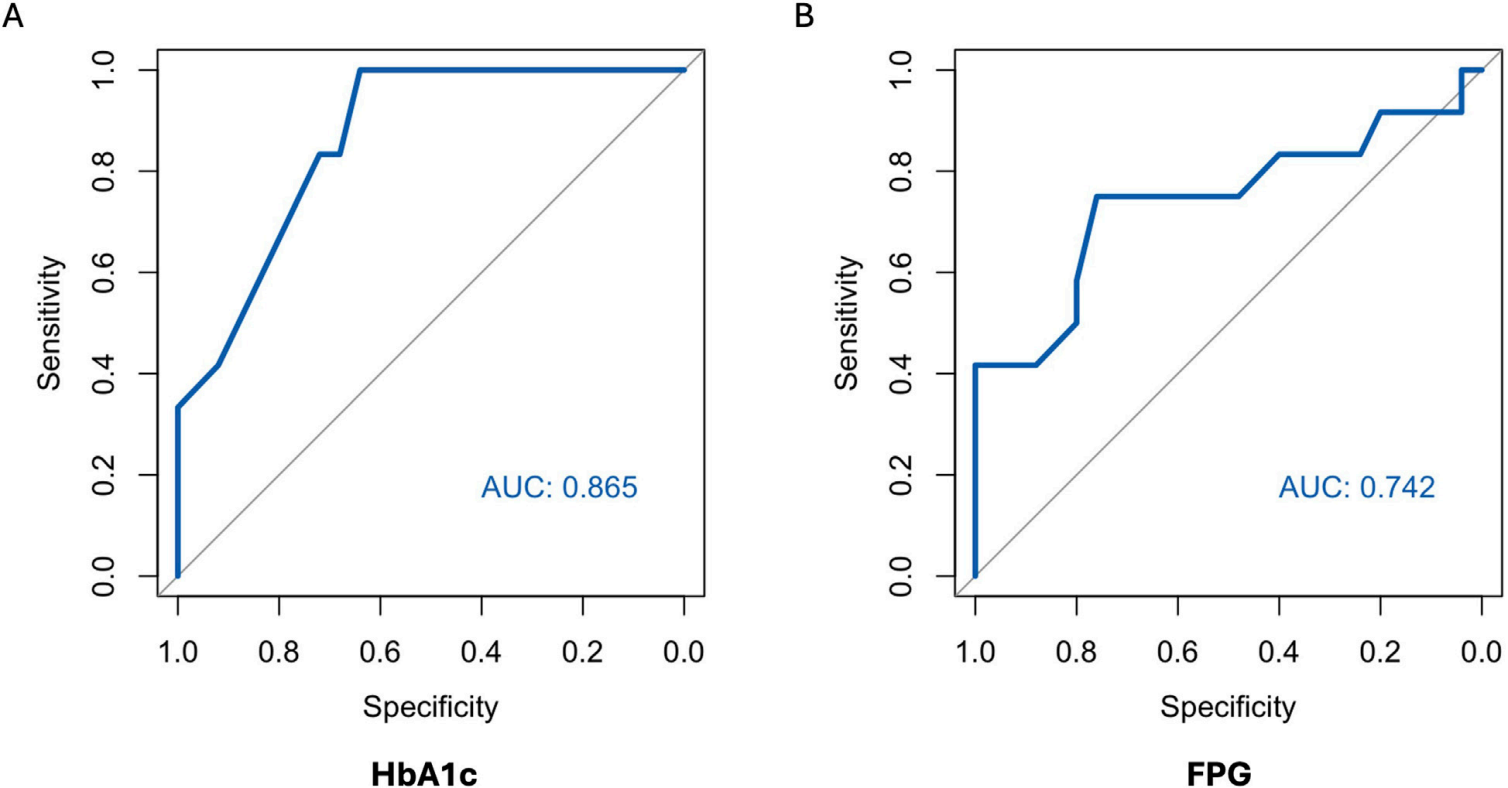
AUC (area under the curve) derived by receiver operating characteristics curve analysis. Diagnosis of IGT vs. NGT with HbA1c **(A)** and FPG **(B)**. Reference test: IGT defined by 2hPG. 2hPG = oGTT-derived 2-h plasma glucose, FPG, fasting plasma glucose; HbA1c, hemoglobin A1c; IGT, impaired glucose tolerance; NGT, normal glucose tolerance.

**FIGURE 6:**
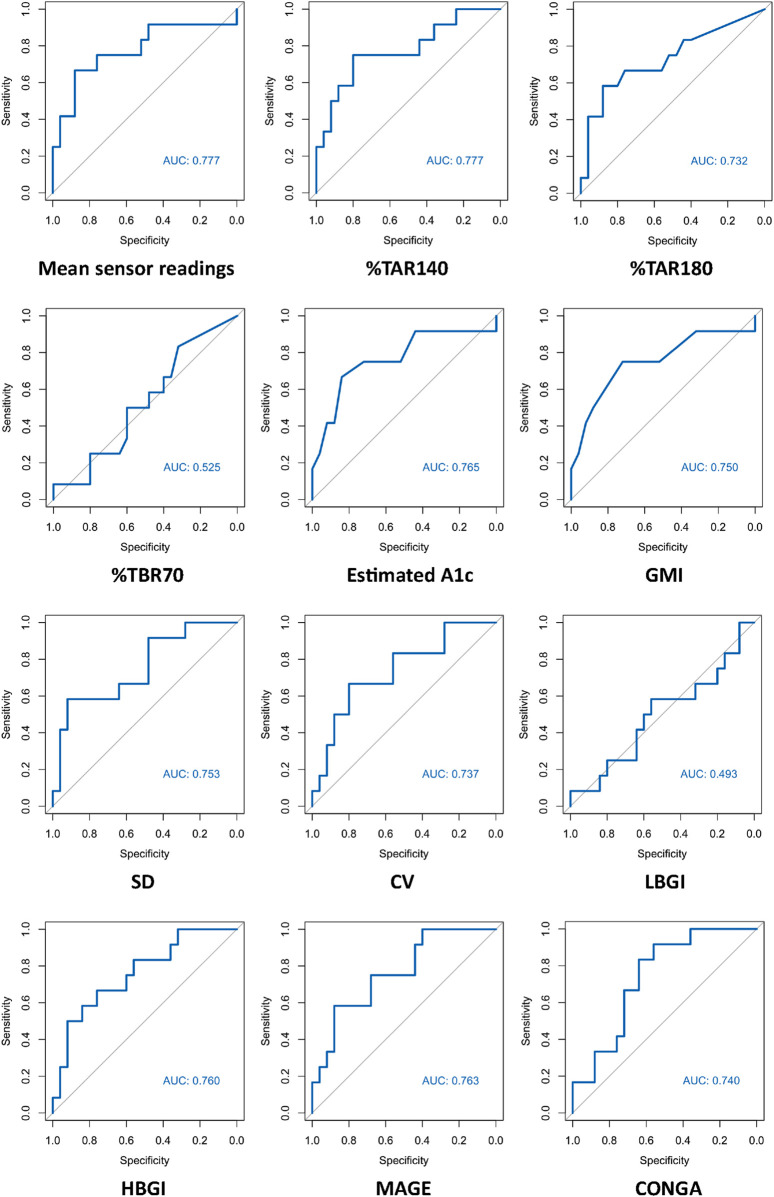
AUC (area under the curve) derived by receiver operating characteristics curve analysis. Diagnosis of IGT vs. NGT with CGM-readouts. Reference test: IGT defined by 2hPG. %TAR, percent of time above range; %TBR, percent of time below range; 2hPG, oral glucose tolerance test (oGTT)-derived 2-h plasma glucose; CONGA, continuous overall net glycemic action; CV, coefficient of variation; GMI, glucose management indicator; HBGI, high blood glucose index; IGT, impaired glucose tolerance; LBGI, low blood glucose index; MAGE, mean amplitude of glycemic excursions; NGT, normal glucose tolerance; SD, standard deviation.

ROC curves of HbA1c, FPG and CGM-readouts for the diagnosis of IGT vs. NGT based on the gold standard 2hPG were plotted (Figures 5, 6) Diagnostic test characteristics were good for HbA1c (ROC-AUC 0.87). FPG and CGM-readouts mean sensor readings, %TAR (140 mg/dL), %TAR (180 mg/dL), estimated A1c, GMI, SD, CV, HBGI, MAGE and CONGA displayed acceptable test characteristics (ROC-AUC 0.74 and 0.78, 0.78, 0.73, 0.77, 0.75, 0.75, 0.74, 0.76, 0.76, and 0.74) while %TBR (70 mg/dL) and LBGI performed poorly (ROC-AUC 0.53 and 0.49) (Table 6).

**TABLE 6:** Diagnosis of IGT vs. NGT.

Laboratory parameters		
	HbA1c	FPG
ROC AUC (CI)	0.87 (0.75–0.98)	0.74 (0.54–0.94)

CGM-readouts						
	Mean Sensor	%TAR (140 mg/dL)	%TAR (180 mg/dL)	%TBR (70 mg/dL)	Estimated A1c	GMI
ROC AUC (CI)	0.78 (0.59–0.96)	0.78 (0.60–0.95)	0.73 (0.54–0.92)	0.53 (0.33–0.72)	0.77 (0.58–0.95)	0.75 (0.56–0.94)

	SD	CV	LBGI	HBGI	MAGE	CONGA
ROC AUC (CI)	0.75 (0.58–0.93)	0.74 (0.56–0.91)	0.49 (0.28–0.70)	0.76 (0.59–0.93)	0.76 (0.60–0.93)	0.74 (0.58–0.90)

%TAR, percent of time above range; %TBR, percent of time below rang; 2hPG, oral glucose tolerance test (oGTT)-derived 2-h plasma glucose; CONGA, continuous overall net glycemic action; CV, coefficient of variation; GMI, glucose management indicator; HBGI, high blood glucose index; IGT, impaired glucose tolerance; LBGI, low blood glucose index; MAGE, mean amplitude of glycemic excursions; NGT, normal glucose tolerance; SD, standard deviation.

Detailed in-sample test characteristics of current ADA-defined HbA1c- and FPG-prediabetes thresholds as well as exploratory screening thresholds of CGM-readouts mean sensor readings and %TAR (140 mg/dL) regarding IGT vs. NGT are provided in Tables 7, 8.

**TABLE 7:** Test characteristics of HbA1c- and FPG-prediabetes thresholds regarding IGT vs. NGT (based on the current criteria of the American Diabetes Association).

	Threshold	Sensitivity	Specificity	PPV	NPV	TP	FN	TN	FP
HbA1c	5.7% (NGSP) 39 mmol/mol (IFCC)	1	0.64 (0.44–0.84)	0.57 (0.46–0.75)	1	12	0	16	9
FPG	100 mg/dL	0.42 (0.17–0.67)	0.96 (0.88–1)	0.83 (0.5–1)	0.77 (0.70–0.86)	5	7	24	1

HbA1c, hemoglobin A1c; FN, false negatives; FP, false positives; FPG, fasting plasma glucose; IFCC, international federation of clinical chemistry and laboratory medicine; NGSP, national glycohemoglobin standardization program; NPV, negative predictive value; PPV, positive predictive value; TN, true negatives; TP, true positives.

**TABLE 8:** Test characteristics of exploratory CGM-screening thresholds regarding IGT vs. NGT. Screening thresholds were calculated for sensitivities directly above and directly below 90%.

Timepoint	Threshold	Sensitivity	Specificity	PPV	NPV	TP	FN	TN	FP
CGM – mean sensor readings	104.7 mg/dL	0.92 (0.75–1)	0.48 (0.28–0.68)	0.46 (0.37–0.58)	0.92 (0.77–1)	11	1	12	13
	105.6 mg/dL	0.83 (0.58–1)	0.52 (0.32–0.72)	0.45 (0.35–0.59)	0.87 (0.71–1)	10	2	13	12
CGM - %TAR (140 mg/dL)	4.4%	0.92 (0.75–1)	0.36 (0.16–0.56)	0.41 (0.32–0.50)	0.90 (0.67–1)	11	1	9	16
	5.3%	0.83 (0.58–1)	0.44 (0.24–0.64)	0.42 (0.32–0.53)	0.85 (0.67–1)	10	2	11	14

%TAR (140 mg/dL), percent of time >140 mg/dL; FN, false negatives; FP, false positives; NPV, negative predictive value; PPV, positive predictive value; TN, true negatives; TP, true positives.

### Feasibility and Tolerability of CGM

Overall, 41 sensors were returned. Three patients displayed recording durations <10 days thus leading to study exclusion. On a scale from 0 (“no discomfort at all”) to 10 (“highest discomfort”), mean patient vote was 1.1, indicating low discomfort. No infectious complications associated to CGM-sensors were noted. 82% of patients (31/38) would have preferred CGM in an unblinded fashion.

## Discussion

In this prospective cross-sectional pilot study of 38 KTR without known preexisting diabetes mellitus (by means of HbA1c or glucose-lowering therapy) one to 5 years after transplantation, prevalence of PTDM and IGT, as defined by the gold standard 2hPG, amounted to 3% and 32% respectively. The major finding of this study is that HbA1c exhibits good diagnostic test characteristics for IGT vs. NGT from years one to five after kidney transplantation. This potentially re-established diagnostic capacity of HbA1c in the stable phase after kidney transplantation, leading to according diagnoses and treatment, could be one explanation for the low PTDM-prevalence in our study. In a large multi-centric prospective study, Porrini et al. had quantified oGTT-based PTDM- and prediabetes-rates from year one to five after kidney transplantation between 21%–34% and 17%–22%, respectively [23]. In our study, maximum Youden’s index was noted for HbA1c 5.7% (NGSP) or 39 mmol/mol (IFCC); at this cut-off sensitivity and specificity regarding IGT were 1.0 and 0.64, respectively. The results of our study are in contrast to those of Kurnikowski et al. [10]. Though showing a progressive improvement over time, HbA1c cut-off of 5.7% (NGSP) or 39 mmol/mol (IFCC) at 2 years still displayed limited diagnostic test characteristics regarding 2hPG (sensitivity 0.55 and specificity 0.82 for IGT) [10]. The discrepancy between our findings might be attributed to the progressive harmonization between HbA1c and the oGTT with time from transplantation. In addition, both studies did not employ confirmatory oGTTs; though the current gold standard for diagnosis of PTDM and prediabetes, limited reproducibility of the OGTT remains a well-known weakness of the test [24]. Since prediabetes, when diagnosed by an oGTT 12 months after transplantation, is an established potentially reversible cardiovascular risk factor [1], our data imply that HbA1c can aid in timely diagnosis and treatment.

Our second major finding is that best-performing CGM-readouts mean sensor readings and %TAR (140 mg/dL) display acceptable test characteristics regarding IGT from years one to five after kidney transplantation (ROC-AUC 0.78 for both). Though not studied extensively, differences in CGM-readouts between non-transplanted oGTT-defined normoglycemic and prediabetic subjects have been described; in the study of Costa et al. mean %TAR (140 mg/dL) was 19% for prediabetic and 13.9% (diabetes high risk group)/3.9% (control group) for normoglycemic patients [25], while in the study of Hanefeld et al. mean %TAR (140 mg/dL) was 13% for prediabetic and 5.7% for normoglycemic patients [26]. Inter-group differences in %TAR (140 mg/dL) were more pronounced in our study (mean: 19% for IGT-vs. 7.7% for NGT-patients). Though in need of prospective validation, this intriguing finding could be a result of immunosuppressive medications (especially steroids) amplifying patient-specific CGM-signatures, thus enhancing discrimination between 2hPG-subgroups over the duration of 14 days CGM.

To our knowledge, this is the first study to assess the diagnostic performance of CGM-readouts compared to traditional glycemic parameters in the stable phase after KTR. Strengths of this study are its prospective design and the use of the oGTT as gold standard (as recommended for clinical practice by the international consensus meeting on PTDM in 2013 [4] and 2022 [5]). The main limitation of the study is its restricted sample size with 38 patients. All patients were Caucasian and a combination of calcineurin inhibitor, mycophenolate and steroids was used for immunosuppression, limiting generalizability to other patient groups or immunosuppressive regimens. Nutritional uptake and physical activity were not assessed.

Our study adds to the existing knowledge around PTDM by highlighting the high prevalence of IGT from years one to five after kidney transplantation and reassessing the role of HbA1c as a reliable parameter for the diagnosis of IGT during this phase. Best-performing CGM-readouts mean sensor readings and %TAR (140 mg/dL) displayed acceptable diagnostic performance. Prospective studies to determine whether CGM-readouts can predict clinically relevant nonglycemic outcomes better than the oGTT in KTR remain of interest.

## Data Availability

The raw data supporting the conclusions of this article will be made available by the authors, without undue reservation.

## Glossary

%TAR (140 mg/dL): percent of time >140 mg/dL
%TAR (180 mg/dL): percent of time >180 mg/dL
%TBR (70 mg/dL): percent of time <70 mg/dL
2hPG: oral glucose tolerance test-derived 2-h plasma glucose
ADA: American Diabetes Association
ADPKD: autosomal dominant polycystic kidney disease
AUC: area under the curve
CGM: continuous glucose monitoring
CI: 95% confidence intervals
CONGA: continuous overall net glycemic action
CV: coefficient of variation
ESKD: end stage kidney disease
FPG: fasting plasma glucose
GMI: glucose management indicator
HbA1c: hemoglobin A1c
HBGI: high blood glucose index
HDL: high density lipoprotein
IFCC: International Federation of Clinical Chemistry and Laboratory Medicine
IGT: impaired glucose tolerance
IQR: interquartile range
KTR: kidney transplant recipient
LBGI: low blood glucose index
LDL: low density lipoprotein
MAGE: mean amplitude of glycemic excursions
NGSP: National Glycohemoglobin Standardization Program
NGT: normal glucose tolerance
oGTT: oral glucose tolerance test
PTDM: post-transplantation diabetes mellitus
ROC: receiver operating characteristic
SD: standard deviation
WHO: World Health Organization
