# Supplementary material for: Continuous Glucose Monitoring for the Diagnosis of Post-Transplantation Diabetes Mellitus and Impaired Glucose Tolerance From Years One to Five After Kidney Transplantation—A Prospective Pilot Study
Source: Transpl Int. 2024 Nov 15;37:13724. doi: 10.3389/ti.2024.13724 (PMC11605328; doi:10.3389/ti.2024.13724)
Supplement: Supplementary file 1 [file Table1.docx]

**Supplementary File**

**S1.** Diagnostic criteria of Post-transplantation Diabetes mellitus (PTDM) and prediabetes based on the current criteria of the American Diabetes Association.

|  | **PTDM** | **Prediabetes** |
| --- | --- | --- |
| *2hPG* | ≥ 200 mg/dL | 140-199 mg/dL |
| *HbA1c* | ≥ 6.5% (NGSP) or 48 mmol/mol (IFCC) | 5.7-6.4 % (NGSP) or 39-47mmol/mol IFCC) |
| *FPG* | ≥ 126 mg/dL | 100-125 mg/dL |

*2hPG=oral glucose tolerance test-derived 2-hour plasma glucose,* FPG=fasting plasma glucose, *HbA1c=hemoglobin A1c, IFCC=International Federation of Clinical Chemistry and Laboratory Medicine, NGSP=National Glycohemoglobin Standardization Program, PTDM=post-transplantation diabetes mellitus*
